# Difructose dianhydride improves intestinal calcium absorption, wound healing, and barrier function

**DOI:** 10.1038/s41598-018-26295-7

**Published:** 2018-05-18

**Authors:** Sang In Lee, In Ho Kim

**Affiliations:** Department of Animal Resource and Science, Dankook University, Cheonan, Chungnam 330–714 Republic of Korea

## Abstract

The gastrointestinal tract (GIT) is critical for nutrient absorption and is an important barrier against harmful pathogens and antigens. Difructose anhydrides (DFA)-IVs are nondigestible disaccharides that enhance calcium and iron absorption by affecting the intestinal epithelial tissue. However, their effects on intestinal functions are not fully understood. In this study, we performed a feeding trial and found that dietary DFA-IVs improved growth performance, relative breast muscle and liver weight, and digestibility and blood calcium and iron concentrations in broilers. Additionally, dietary DFA-IVs increased expression of genes related to growth in the liver, muscle development, and absorption of calcium and iron in the intestine. *In vitro* experiments revealed that DFA-IV treatment improved intestinal wound-healing (migration, proliferation, and differentiation) after lipopolysaccharide (LPS) challenge in small intestinal epithelial cells. Furthermore, DFA-IV treatment enhanced the intestinal barrier function, which increased the transepithelial electrical resistance (TEER) and decreased the permeability of fluorescein isothiocyanate-dextran (FD-4) after LPS challenge in small intestinal epithelial cells. Collectively, these data indicate that DFA-IV could be used as a feed additive to enhance calcium and iron absorption by affecting the intestinal wound healing and permeability. This study may help improve our understanding of the molecular effects of DFA-IV on the intestine.

## Introduction

The gastrointestinal tract (GIT) plays critical roles in the digestion and absorption of nutrients and the maintenance of the host barrier function against harmful pathogens and antigens^1,2^. It is important to maintain appropriate intestinal functions because malfunction of the intestine is directly related to livestock health and growth^3^. However, the recent ban on antibiotic use in feedstuffs has caused numerous problems with growth performance and intestinal health. To overcome the challenges associated with the ban of antibiotics in feedstuffs, a number of alternatives such as probiotics, prebiotics, organic acids, and plant extracts have been proposed^4^.

Among the alternative to antibiotics, difructose anhydrides (DFAs) prebiotics are cyclic nondigestible disaccharides that consist of two fructose units linked at their reducing carbons. Four kinds of DFAs have been reported, DFA I, III, IV, and V based on the degradation of inulin and levan by the microbial enzymes, inulin and levan fructotransferase, respectively^5,6^. DFA-III and IV were reported to enhance the absorption of minerals, particularly calcium and iron in *in vivo* and *vitro* experiments^7–11^. DFA administration directly increased the numbers of health-promoting bacteria and decreased harmful bacteria in the host GIT^12^. Moreover, DFAs stimulate short-chain fatty acids (SCFAs) including acetic acid, which may alter the intestinal microbiota towards a healthier composition^12,13^. It has been reported that DFAs directly affect the intestinal epithelial tissue and activate the permeability and intercellular passage through tight junctions, promoting calcium absorption in the small and large intestines^14^. However, little is known about the effect of DFA-IV on the physiological function of the small intestine.

It would be expedient to study the effect of DFA-IV on the intestinal epithelium and the integrity of the intestine. Therefore, in the present study, we evaluated the effects of DFA-IV, as a prebiotic, on the growth performance, relative breast muscle and liver weight, and digestibility and blood concentration of calcium and iron. Furthermore, we analysed the expression of genes related to growth in the liver, muscle development, and, intestinal absorption of calcium and iron in the broilers. Moreover, we investigated the effects of DFA-IV on intestinal wound healing, by evaluating migration, proliferation, and differentiation. In addition, we examined the intestinal barrier function by confirming the transepithelial electrical resistance (TEER) and permeability of FD-4 after lipopolysaccharide (LPS) challenge of small intestinal epithelial cells.

## Results

### DFA-IV enhanced growth performance and relative organ weight of muscle

**Figure 1:**
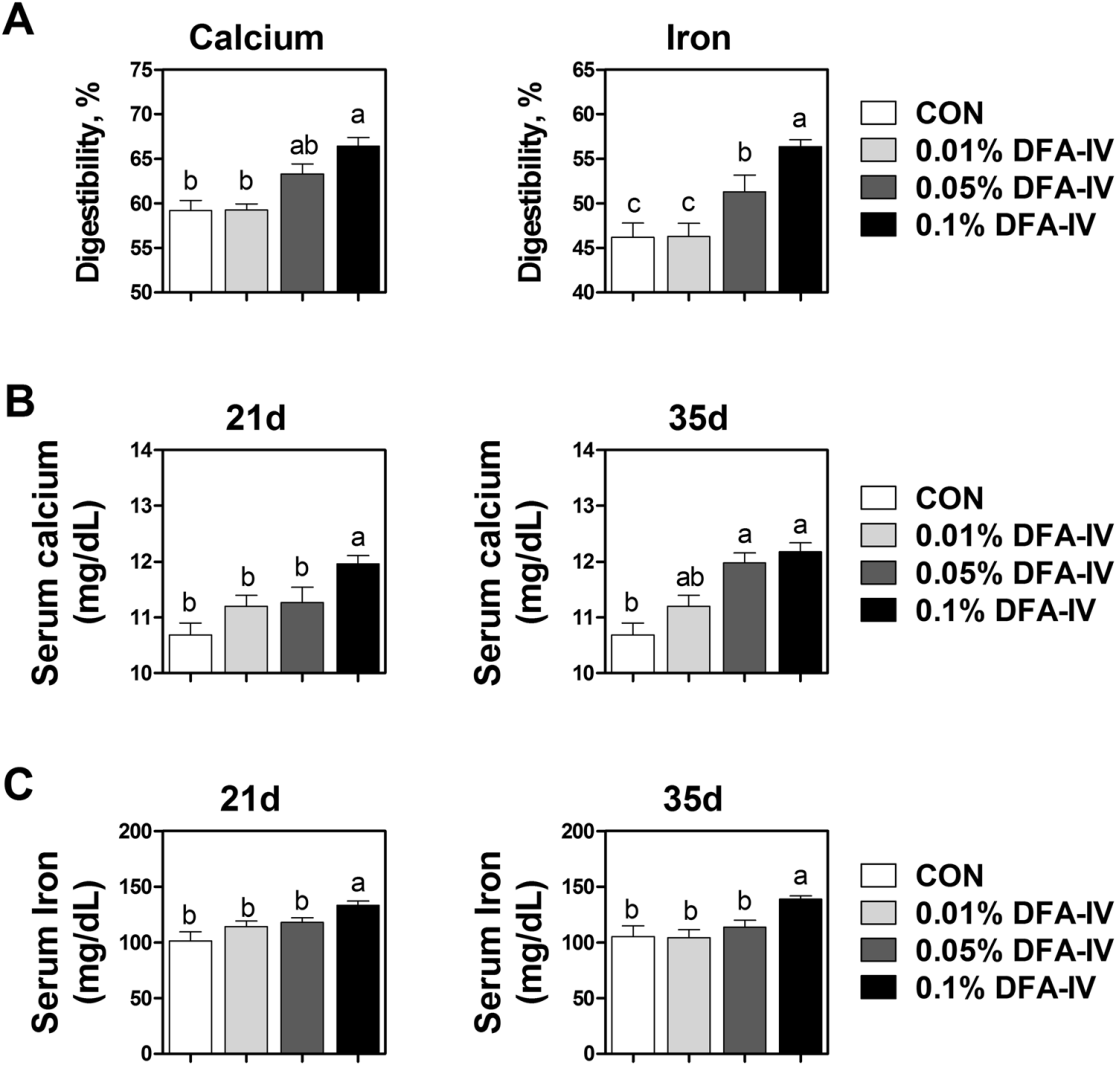
Effect of dietary difructose anhydride (DFA)-IV supplementation on growth performance of broilers *in vivo*. Broilers were randomly allocated to four groups: CON, corn-soybean meal-based control; 0.01% DFA-IV, corn-soybean meal-based control plus 0.01% DFA-IV; 0.05% DFA-IV, corn-soybean meal-based control plus 0.05% DFA-IV; and 0.1% DFA-IV, corn-soybean meal-based control plus 0.1% DFA-IV. (**A**) ADG, (**B**) ADFI, and (**C**) FCR of broilers in four groups over time during experiments. Diets were fed in two phases (phase I and II from d 0 to 21, and d 21 to 35, respectively, and phase I + II from d 0 to 35). ^a,b^p < 0.05, between treatments based on Duncan’s multiple range tests. Error bars indicate standard errors (SEs) of analyses (n = 12). ADG, average daily gain; ADFI, average daily feed intake; FCR, feed conversion ratio.

**Figure 2:**
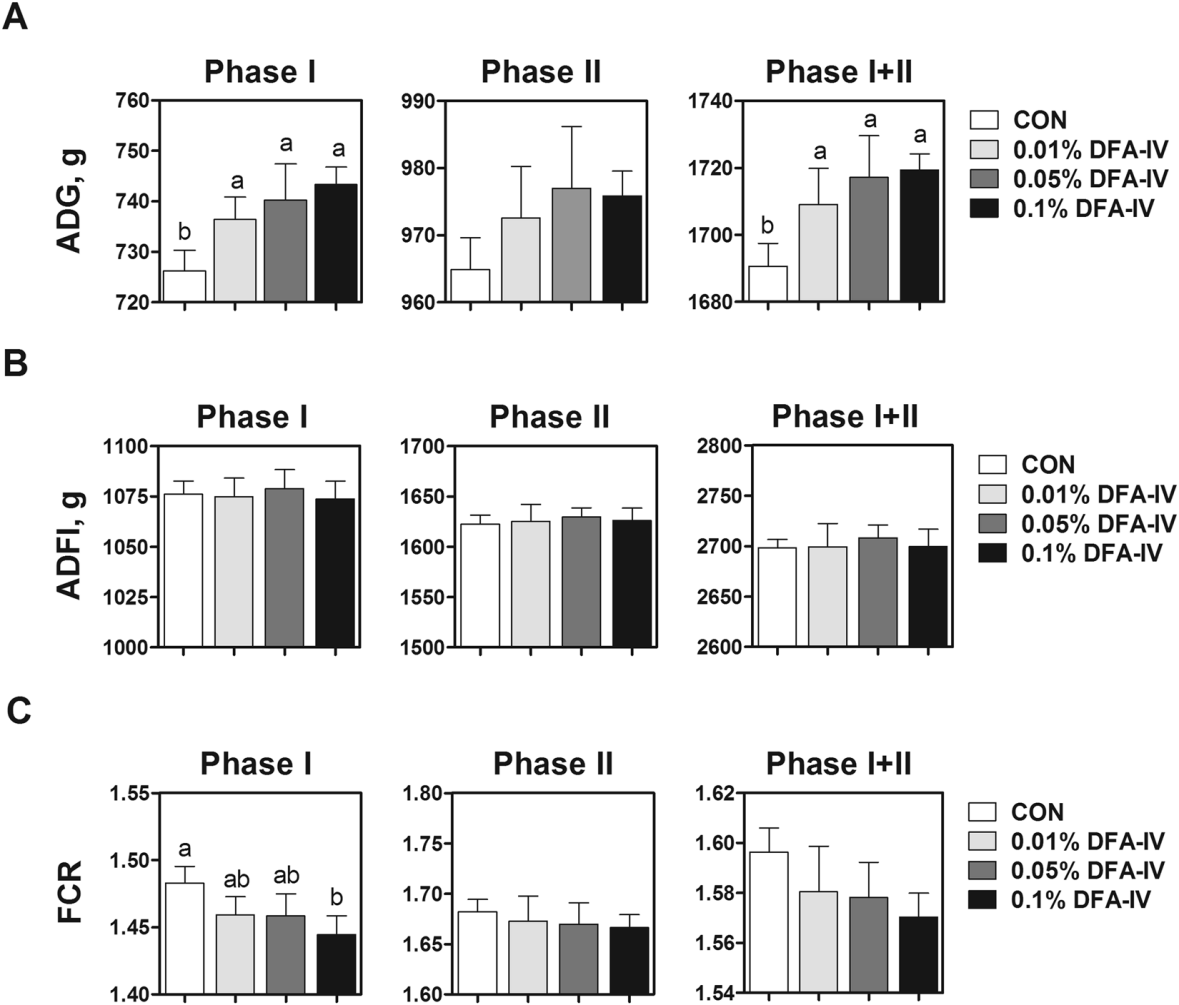
Effect of dietary difructose anhydride (DFA)-IV supplementation on relative breast muscle and liver weight of broilers *in vivo*. ^a,b^p < 0.05, between treatments based on Duncan’s multiple range tests. Error bars indicate standard error (SE) of analyses (n = 10).

The average daily gain (ADG) was enhanced by DFA-IV (0.01, 0.05, and 0.1%) supplementation in feeding phases I and I + II compared with the control (Fig. 1A). DFA-IV supplementations caused no substantial differences in the average daily feed intake (ADFI) compared with the control (Fig. 1B). The 0.1% DFA-IV supplementation decreased the feed conversion ratio (FCR) in phase I compared with the control (Fig. 1C). Furthermore, 0.05 and 0.1% DFA-IV supplementation effectively increased the relative breast muscle weight compared with the control (Fig. 2). However, DFA-IV supplementation showed no marked differences in the relative liver weight compared with the control (Fig. 2).

### DFA-IV increased expression of genes related to growth and muscle development

**Figure 3:**
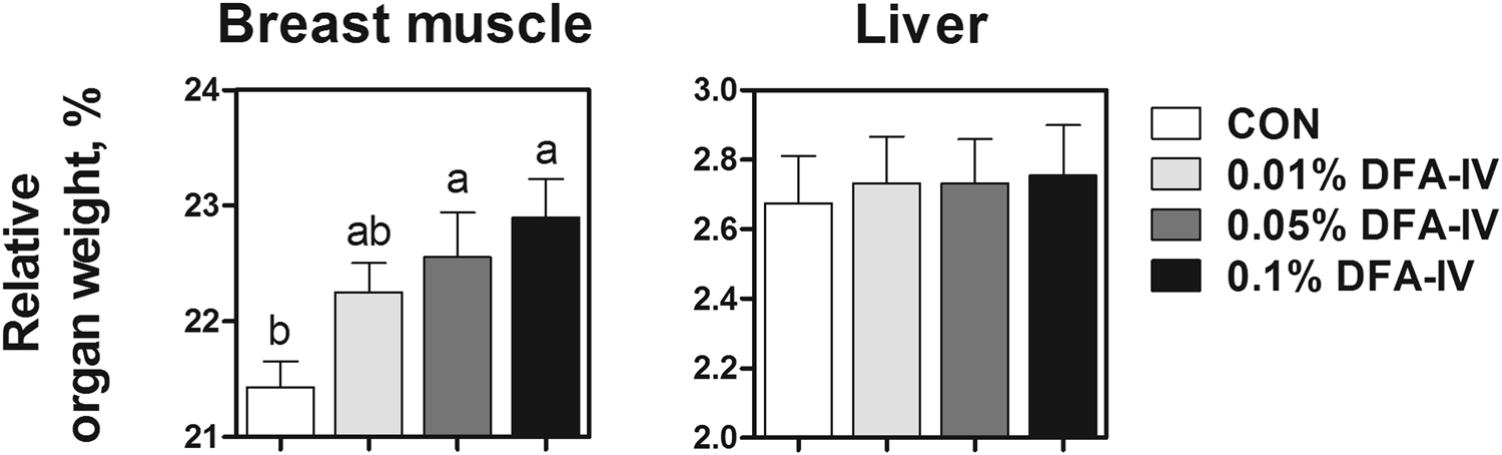
Quantitative gene expression of growth- and muscle-related genes following dietary difructose anhydride (DFA)-IV supplementation in broilers. (**A**) Expression pattern of growth-related genes (insulin-like growth factor receptor 1 [*IGFR1*], *IGFR2*, growth factor receptor [*GHR*], and leptin [*LEPR*]) in liver following feeding with DFA-IVs. (**B**) Expression pattern of muscle development-related genes (myostatin, [*MSTN*], myogenic differentiation 1 [*MYOD1*], myogenin [*MYOG*], and myogenic factor 4 [*MYF4*]) in muscle following feeding with DFA-IVs. Quantitative reverse transcription polymerase chain reaction (qRT-PCR) data were normalised relative to the expression of the *GAPDH* as an endogenous control and calculated using the 2^ΔΔCt^ method, where ΔΔCt = (Ct of the target gene − Ct of *GAPDH*) treatment − (Ct of the target gene − Ct of *GAPDH*) control. Significant differences between control and treatment groups were indicated as *p < 0.05 and **p < 0.01. Error bars indicate standard errors of triplicate analyses. GAPDH, glyceraldehyde 3-phosphate dehydrogenase.

To confirm whether DFA-IV enhanced growth performance and relative breast muscle weight, we examined its effects on the expression pattern of genes related to these phenomena in the liver and breast muscle respectively. DFA-IV significantly increased the gene expression of insulin growth factor 1 receptor (*IGF1R*, p < 0.01), *IGF2R* (p < 0.05), leptin receptor (*LEPR*, p < 0.05), and growth hormone receptor (*GHR*, p < 0.05) in the liver (Fig. 3A). DFA-IV significantly decreased the gene expression of myostatin (*MSTN*, p < 0.01) and increased that of myogenic differentiation 1 (*MYOD1*, p < 0.01), myogenin (*MYOG*, p < 0.05), and myogenic factor 5 (*MYF5*, p < 0.05) in breast muscle (Fig. 3B).

### DFA-IV improved digestibility and blood concentration of calcium and iron

**Figure 4:**
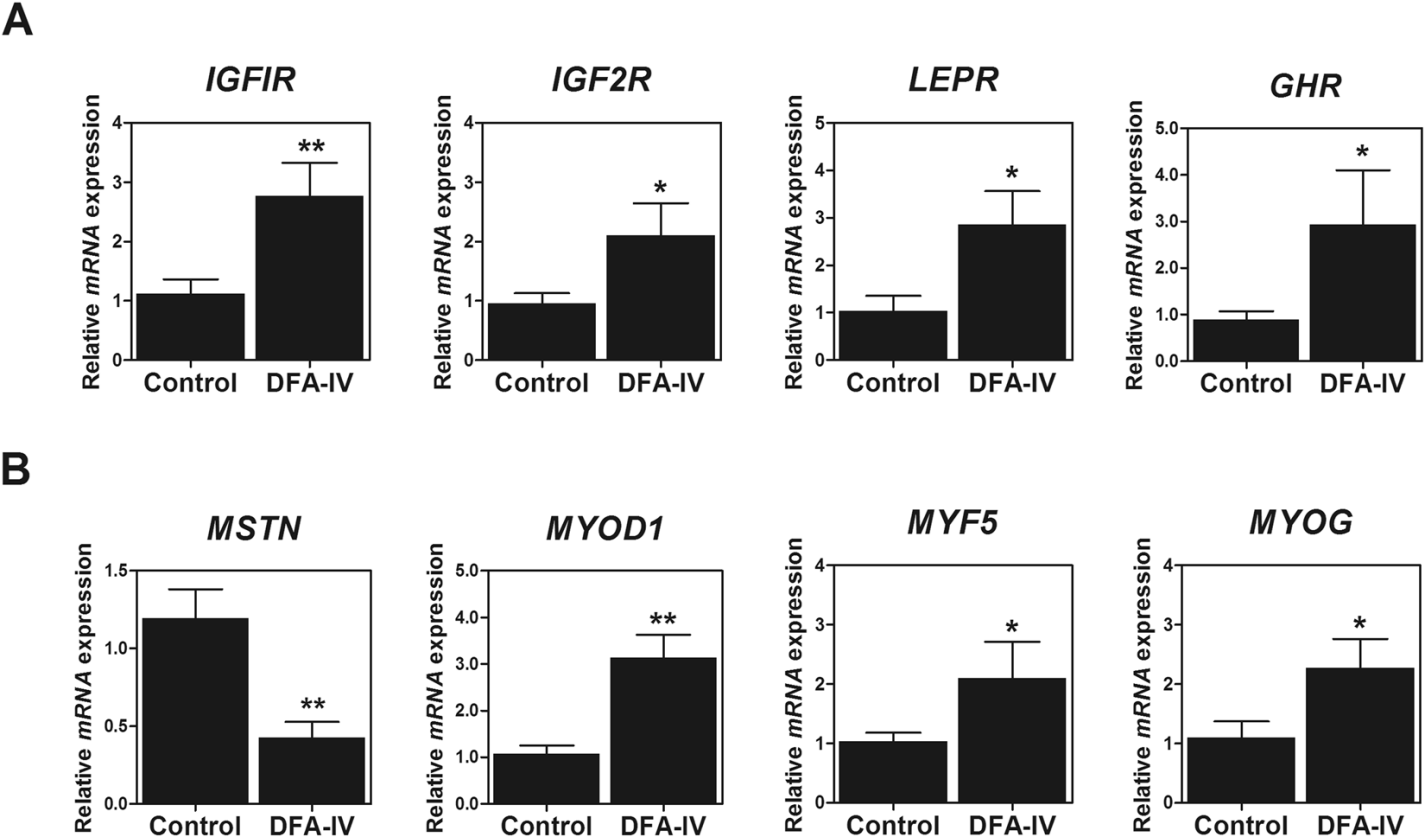
Effect of dietary difructose anhydride (DFA)-IVs supplementation on (**A**) digestibility and (**B** and **C**) blood concentration of calcium and iron of broiler at 21 and 35d *in vivo*. ^a,b,c^p < 0.05, between treatments based on Duncan’s multiple range tests. Error bars indicate standard error (SE) of analyses (n = 10).

To investigate the effect of DFA-IV on calcium and iron absorption, their concentration and digestibility were analysed. Supplementation with 0.1% DFA-IV markedly improved the digestibility of calcium compared with the untreated control (Fig. 4A). Additionally, 0.05 and 0.1% DFA-IV supplementation improved the digestibility of iron compared with the untreated control. Serum calcium concentration was increased by 0.1% DFA-IV supplementation on d 21 and 0.05 and 0.1% DFA-IV supplementation on d 35 (Fig. 4B). Supplementation with 0.1% DFA-IV markedly improved the serum iron concentration compared with the untreated control (Fig. 4C).

### DFA-IV treatment increased expression of genes related to calcium absorption in small intestine

**Figure 5:**
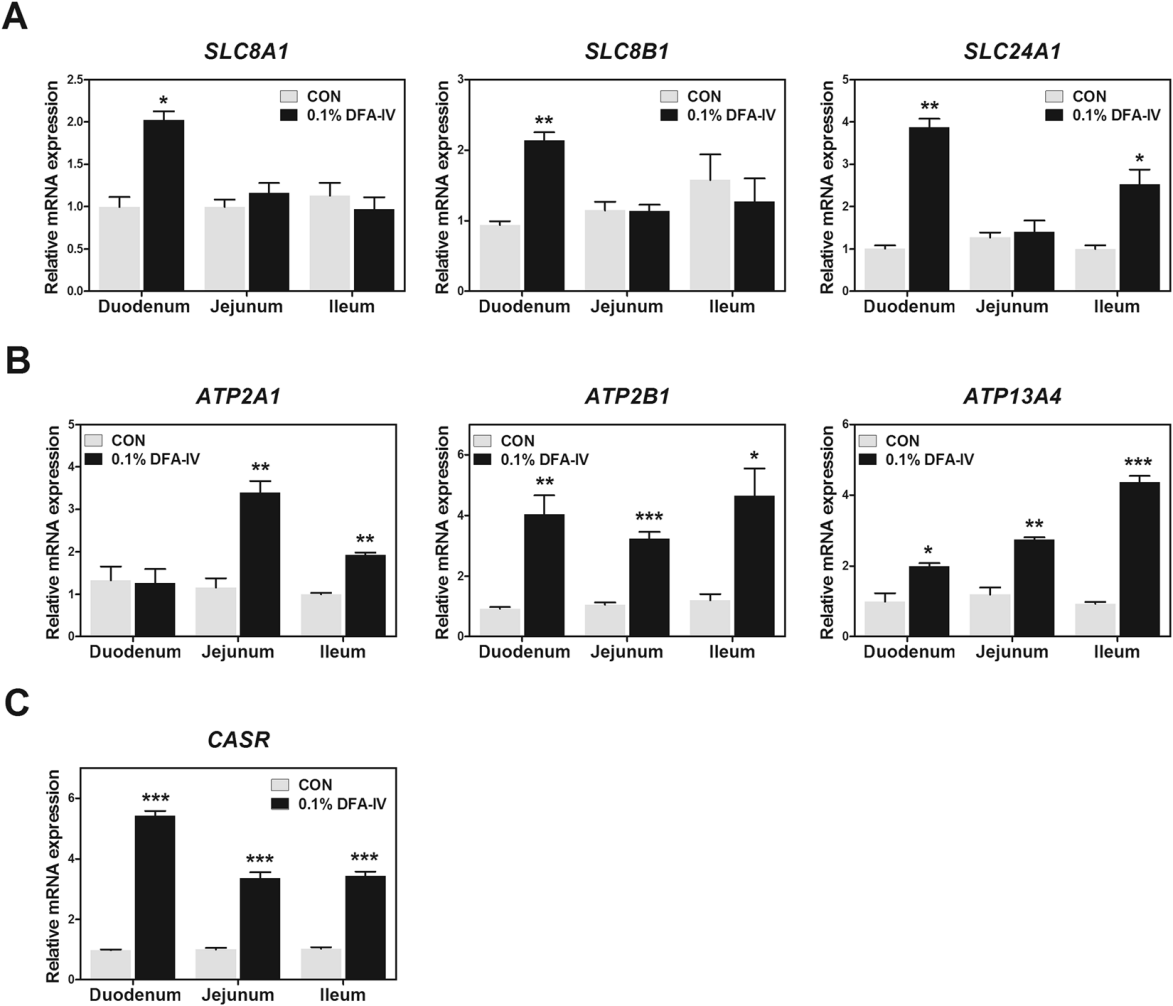
Quantitative gene expression of calcium absorption related genes by dietary 0.1% difructose anhydride (DFA)-IV supplementation on broilers. (**A**) Expression pattern of calcium carrier proteins (solute carrier family 8 sodium/calcium exchanger member 1 [*SLC8A1*, *SLC8B1*, and *SLC24A1*]) in duodenum, jejunum, and ileum following feeding with 0.1% DFA-IV. (**B**) Expression pattern of ATPase (ATPase, Ca^++^ transporting, cardiac muscle fast twitch 1 [*ATP2A1*, *ATP2B1*, and *ATP13A4*]) in duodenum, jejunum, and ileum following feeding with 0.1% DFA-IV. (**C**) Expression pattern of calcium-sensing receptor 1, *CASP* in duodenum, jejunum, and ileum following feeding with 0.1% DFA-IV. The quantitative reverse transcription polymerase chain reaction (qRT-PCR) data were normalised relative to the expression of the *GAPDH* as an endogenous control and calculated using the 2^ΔΔCt^ method, where ΔΔCt = (Ct of the target gene − Ct of *GAPDH*) treatment − (Ct of the target gene − Ct of *GAPDH*) control. Significant differences between control and treatment groups were indicated as *p < 0.05, **p < 0.01, and ***p < 0.001. Error bars indicate the standard errors of triplicate analyses. GAPDH, glyceraldehyde 3-phosphate dehydrogenase.

To confirm whether DFA-IV supplementation increased calcium and iron absorption, we examined its effects on the expression pattern of genes related to calcium transport, ATPase, and calcium channels in the duodenum, jejunum, and ileum. DFA-IV significantly increased the expression of solute carrier family 8 member 1 (*SLC8A1*, *SLC8B1* [both p < 0.05], and *SLC24A1* [p < 0.01]) in the duodenum (Fig. 5A) while *SLC24A1* expression was also increased in the ileum (p < 0.05).

DFA-IV significantly increased the gene expression of ATPase, Ca^++^ transporting, cardiac muscle, fast twitch 1 (*APT2A1*, p < 0.01) in the jejunum and ileum (Fig. 5B). *ATP2B1* expression was increased in the duodenum, jejunum (both p < 0.001), and ileum (p < 0.05) following DFA-IV supplementation. DFA-IV also significantly increased the expression of *APT13A4* in the duodenum (p < 0.05), jejunum, and ileum (both p < 0.001). The calcium sensing receptor (*CARR*, p < 0.001) expression was increased in the duodenum, jejunum, and ileum by DFA-IV supplementation (Fig. 5C).

**Figure 6:**
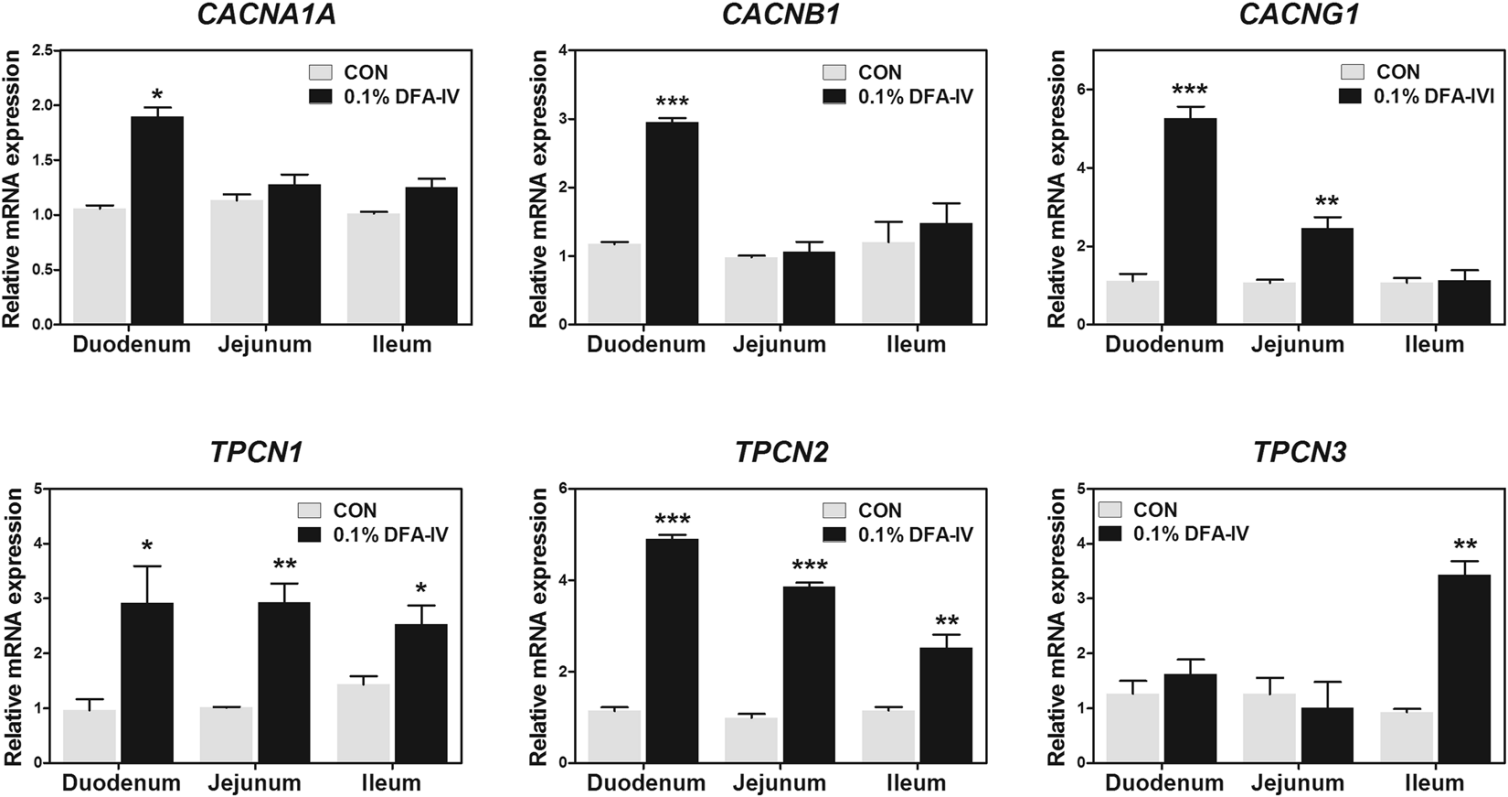
Quantitative gene expression of calcium channel-related genes following dietary 0.1% difructose anhydride (DFA-IV) supplementation in broilers. (**A**) Expression pattern of calcium channels (calcium channel, voltage-dependent, P/Q type, alpha 1 A subunit [*CACNA14*, *CACNB1*, and *CACNG1*]) in duodenum, jejunum, and ileum following feeding with 0.1% DFA-IV. (**B**) Expression pattern of two-pore calcium channels (two-pore calcium channel 3, [*TPCN1*, *TPCN2*, and *TPCN3*]) in duodenum, jejunum, and ileum following feeding with 0.1% DFA-IV. Quantitative reverse transcription polymerase chain reaction (qRT-PCR) data were normalised relative to the expression of the *GAPDH* as an endogenous control and calculated using the 2^ΔΔCt^ method, where ΔΔCt = (Ct of the target gene − Ct of *GAPDH*) treatment − (Ct of the target gene − Ct of *GAPDH*) control. Significant differences between control and treatment groups were indicated as *p < 0.05, **p < 0.01, and ***p < 0.001. Error bars indicate the standard errors of triplicate analyses. GAPDH, glyceraldehyde 3-phosphate dehydrogenase.

DFA-IV significantly increased the expression of calcium channel, voltage-dependent, P/Q type, alpha 1 A subunit (*CACNA1A*, p < 0.05) and *CACNB1* (p < 0.001) in the duodenum (Fig. 6). The CAC, gamma subunit 1 (*CACNG1*) expression was increased in the duodenum (p < 0.001) and jejunum (p < 0.01) by DFA-IV supplementation. DFA-IV significantly increased the expression of the two pore segment channel 1 (*TPCN1*) in the duodenum (p < 0.05), jejunum (p < 0.01), and ileum (p < 0.05), as well as *TPCN2* expression in the duodenum, jejunum (both p < 0.001), and ileum (p < 0.05) while *TPCN3* expression also increased the in the ileum (p < 0.01).

### DFA-IV treatment improved intestinal wound healing

**Figure 7:**
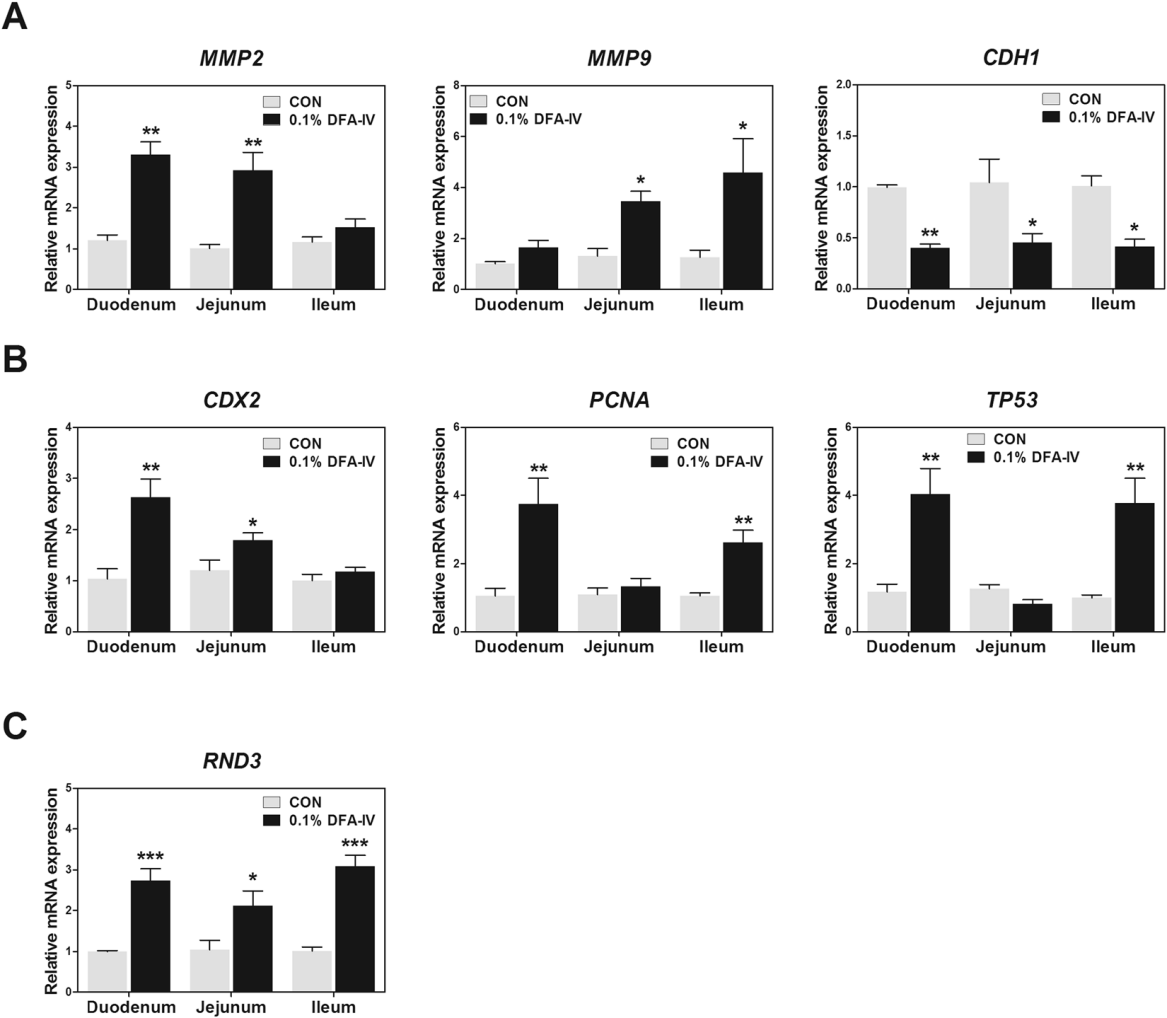
Quantitative gene expression of intestinal wound healing related genes following dietary 0.1% difructose anhydride (DFA)-IV supplementation in broilers. (**A**) Expression pattern of migration-related genes (*MMP2*, *MMP9*, and *CDH1*) in duodenum, jejunum, and ileum following feeding with 0.1% DFA-IV. (**B**) Expression pattern of proliferation-related genes (*CDX1*, *PCNA*, and *TP53*) in duodenum, jejunum, and ileum following feeding with 0.1% DFA-IV. (**C**) Expression pattern of the differentiation-related gene (*RND3*) in duodenum, jejunum, and ileum following feeding with 0.1% DFA-IV. Quantitative reverse transcription polymerase chain reaction (qRT-PCR) data were normalised to expression of *GAPDH* as the endogenous control and calculated using the 2^ΔΔCt^ method, where ΔΔCt = (Ct of the target gene − Ct of *GAPDH*) treatment − (Ct of the target gene − Ct of *GAPDH*) control. Significant differences between control and treatment groups were indicated as *p < 0.05, **p < 0.01, and ***p < 0.001. Error bars indicate the standard errors of triplicate analyses. *MMP*, matrix metallopeptidase; *CDH1*, cadherin 1; *GAPDH*, glyceraldehyde 3-phosphate dehydrogenase, *CDX*, caudal type homeobox; *PCNA*, proliferating cell nuclear antigen; *TP53*, tumour protein p53.

To investigate whether the DFA-IV enhances intestinal wound healing, we analysed the expression pattern of related genes that modulate cell migration, proliferation, and differentiation. The effect of DFA-IV supplementation on the expression of genes related to migration in the small intestine is shown in Fig. 7A. DFA-IV significantly increased the expression of matrix metallopeptidase 2 (*MMP2*, p < 0.01) in the duodenum and jejunum and *MMP9* (p < 0.05) in the jejunum and ileum. Cadherin 1 (*CDH1*) expression was decreased in the duodenum (p < 0.01), jejunum, and ileum (both p < 0.05) following DFA-IV supplementation.

The effect of DFA-IV supplementation on the expression of genes related to small intestinal cell proliferation is shown in Fig. 7B. DFA-IV significantly increased the expression of caudal type homeobox 2 (*CDX2*) in the duodenum (p < 0.01) and jejunum (p < 0.05), and proliferating cell nuclear antigen (*PCNA*, p < 0.01) in the duodenum and ileum. The tumour protein p53 (*TP53*, p < 0.01) expression was increased in the duodenum and ileum by DFA-IV supplementation. The effect of DFA-IV supplementation on the expression of genes related to small intestinal cell differentiation is shown in Fig. 7C. DFA-IV significantly increased the expression of Rho family GTPase 3 (*RND3*) in the duodenum (p < 0.001), jejunum (p < 0.05), and ileum (p < 0.001).

**Figure 8:**
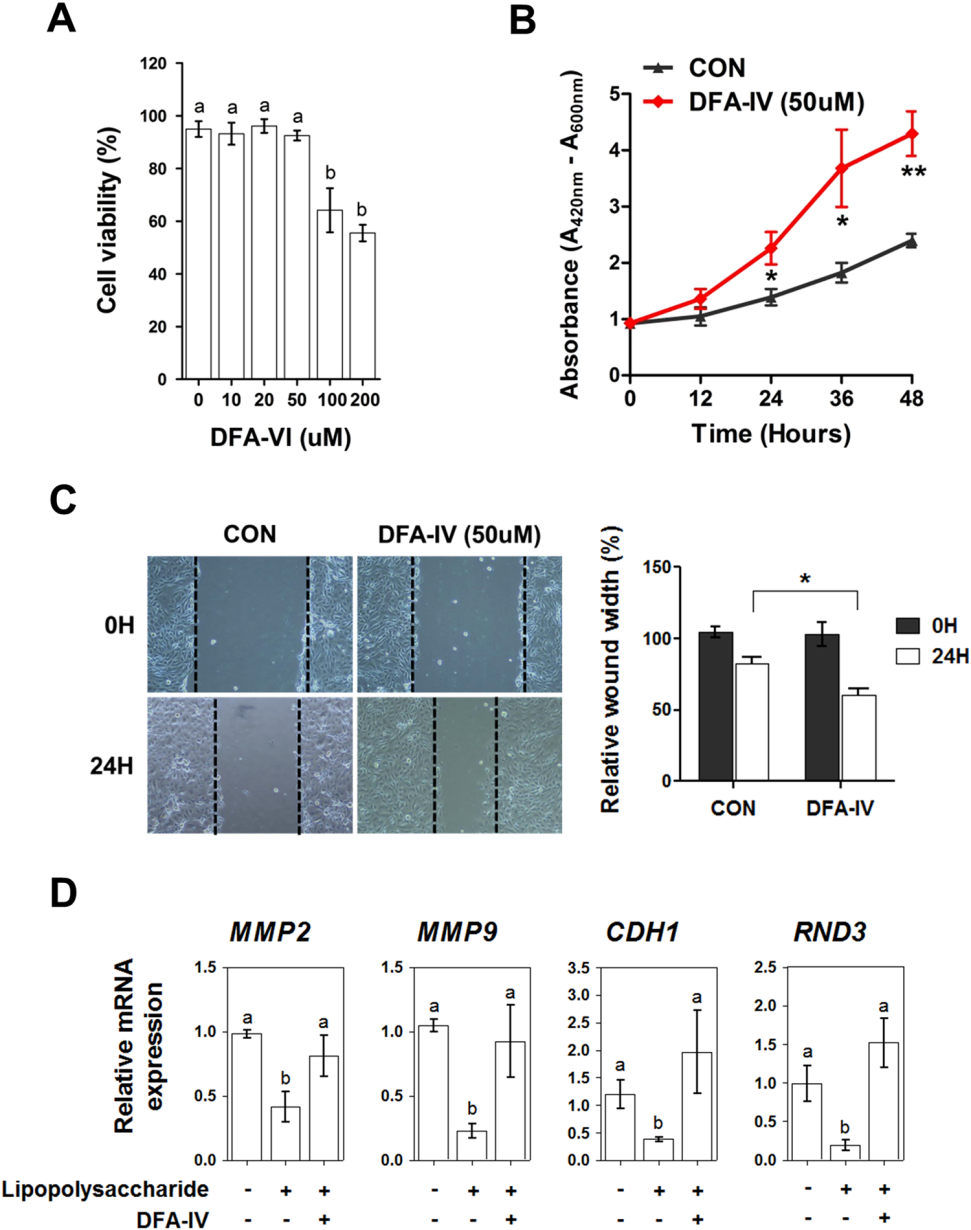
Difructose anhydride (DFA)-IV regulated intestinal wound healing *in vitro*. (**A**) Cytotoxicity of DFA-IV in IPEC-J2 cells. Cell viability was determined using MTT assays. IPEC-J2 cells were incubated with DFA-IV (0–200 µM) for 24 h. ^a,b^p < 0.05, between treatments based on Duncan’s multiple range tests. Error bars indicate standard errors (SEs) of analyses (n = 3). (**B**) The number of viable cells was determined 12, 24, 36, and 48 h after treatment with DFA-IV (50 μM) using WST-1 assays (n = 5). Significant differences between control and treatment groups are indicated as **P < 0.01 and *P < 0.05. (**C**) Effects of DFA-IV (50 μM) on migration in IPEC-J2 cells at 24 h (n = 3). Significant differences between control and treatment groups are indicated as *P < 0.05. (**D**) Expression of wound healing-related genes, such as *MMP2*, *MMP9*, *CDH1*, and *RND3*, analysed with or without DFA-IV (50 μM) after lipopolysaccharide (LPS) challenge in IPEC-J2 cells using real-time polymerase chain reaction (PCR). Quantitative reverse transcription (qRT)-PCR data were normalised to expression of *GAPDH* as an endogenous control and calculated using the 2^ΔΔCt^ method, where ΔΔCt = (Ct of the target gene − Ct of *GAPDH*) treatment − (Ct of the target gene − Ct of *GAPDH*) control. ^a,b^p < 0.05 between treatments based on Duncan’s multiple range tests. Error bars indicate standard error (SE, n = 3). MMP, matrix metallopeptidase; CDH1, cadherin 1; RND3, Rho family GTPase 3; MTT, 3-(4,5-dimethylthiazol-2-yl)-2,5-diphenyltetrazolium bromide; WTS, water-soluble tetrazolium.

To investigate whether DFA-IV treatment affects intestinal wound healing *in vitro*, we determined the appropriate dose of DFA-IV in IPEC-J2 cells by monitoring their viability. Pre-treatment with 100 and 200 µM DFA-IV for 24 h decreased the viability of IPEC-J2 cells (Fig. 8A). On the basis of these results, DFA-IV at 50 µM was considered safe and used for subsequent experiments. Furthermore, DFA-IV treatment stimulated IPEC-J2 cell growth in a time-dependent manner and significantly reduced wound width in scratch assays in IPEC-J2 cells (Fig. 8B and C). In the LPS-challenged IPEC-J2 cells, the expression of MMP2, MMP4, CDH1, and RND3 was significantly lower than that in the control cells. DFA-IV treatment significantly increased the expression of MMP2, MMP4, CDH1, and RND3 in LPS-challenged IPEC-J2 cells (p < 0.05, Fig. 8D).

### DFA-IV treatment improved intestinal barrier function

**Figure 9:**
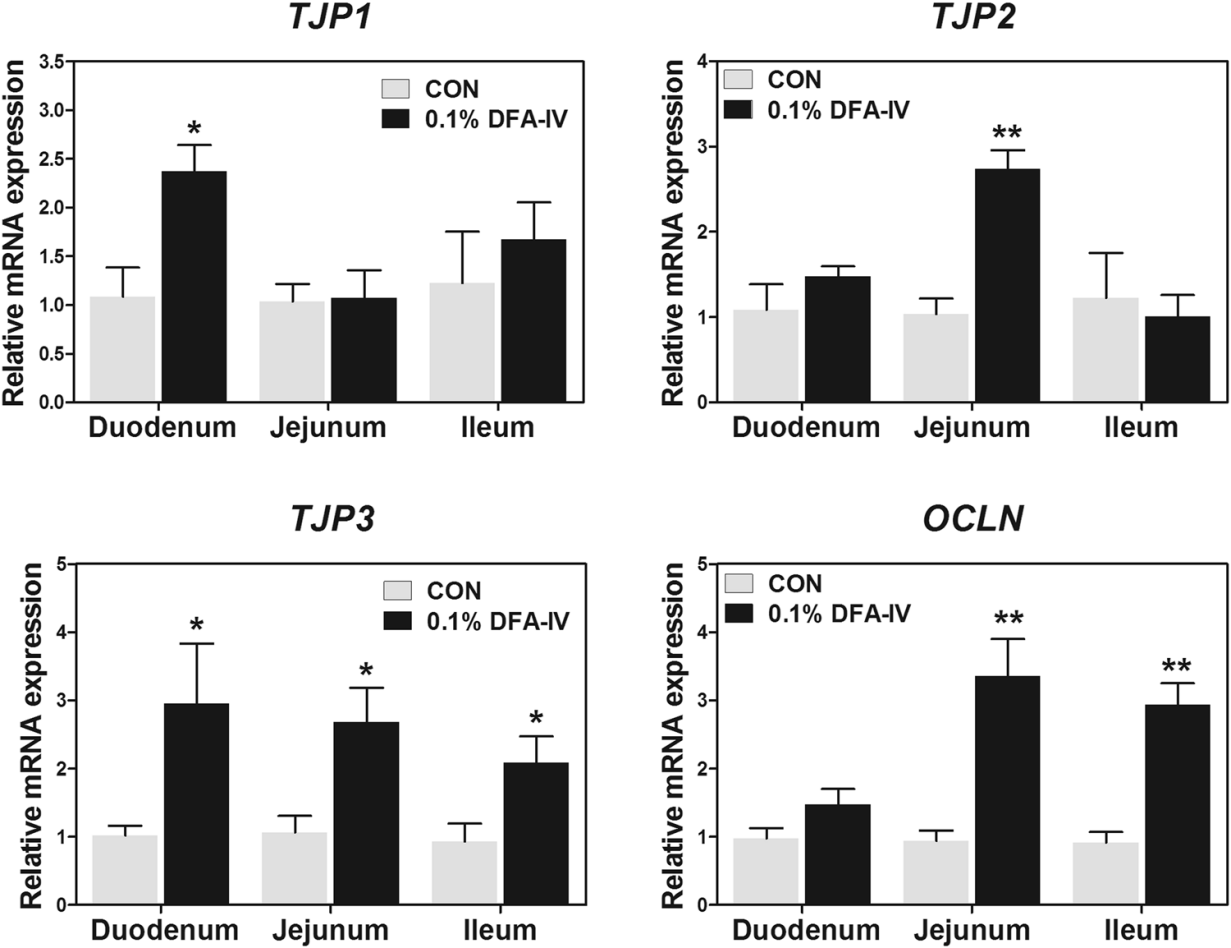
Quantitative gene expression of intestinal barrier function related genes following dietary 0.1% difructose anhydride (DFA)-IV supplementation on broilers. Expression pattern of tight junction protein (*TJP1*, *TJP2*, *TJP3*, and *OCLN*) in duodenum, jejunum, and ileum following feeding with 0.1% DFA-IV. Quantitative reverse transcription polymerase chain reaction (qRT-PCR) data were normalised to expression of *GAPDH* as an endogenous control and calculated using the 2^ΔΔCt^ method, where ΔΔCt = (Ct of the target gene − Ct of *GAPDH*) treatment − (Ct of the target gene − Ct of *GAPDH*) control. Significant differences between control and treatment groups were indicated as *p < 0.05 and **p < 0.01. Error bars indicate the standard errors of triplicate analyses. OCLN, occludin.

To investigate whether the DFA-IV enhances the intestinal barrier function, we evaluated the expression pattern of genes related to tight junctions in the small intestine. The tight junction protein 1 (*TJP1*) and *TJP2* (p < 0.01 and p < 0.05) expression was increased in the duodenum and jejunum, respectively by DFA-IV supplementation (Fig. 9). DFA-IV increased the expression of *TJP3* (p < 0.05) in the duodenum, jejunum, and ileum while that of occludin1 (*OCLN*, p < 0.01) was increased in the jejunum and ileum by DFA-IV supplementation.

**Figure 10:**
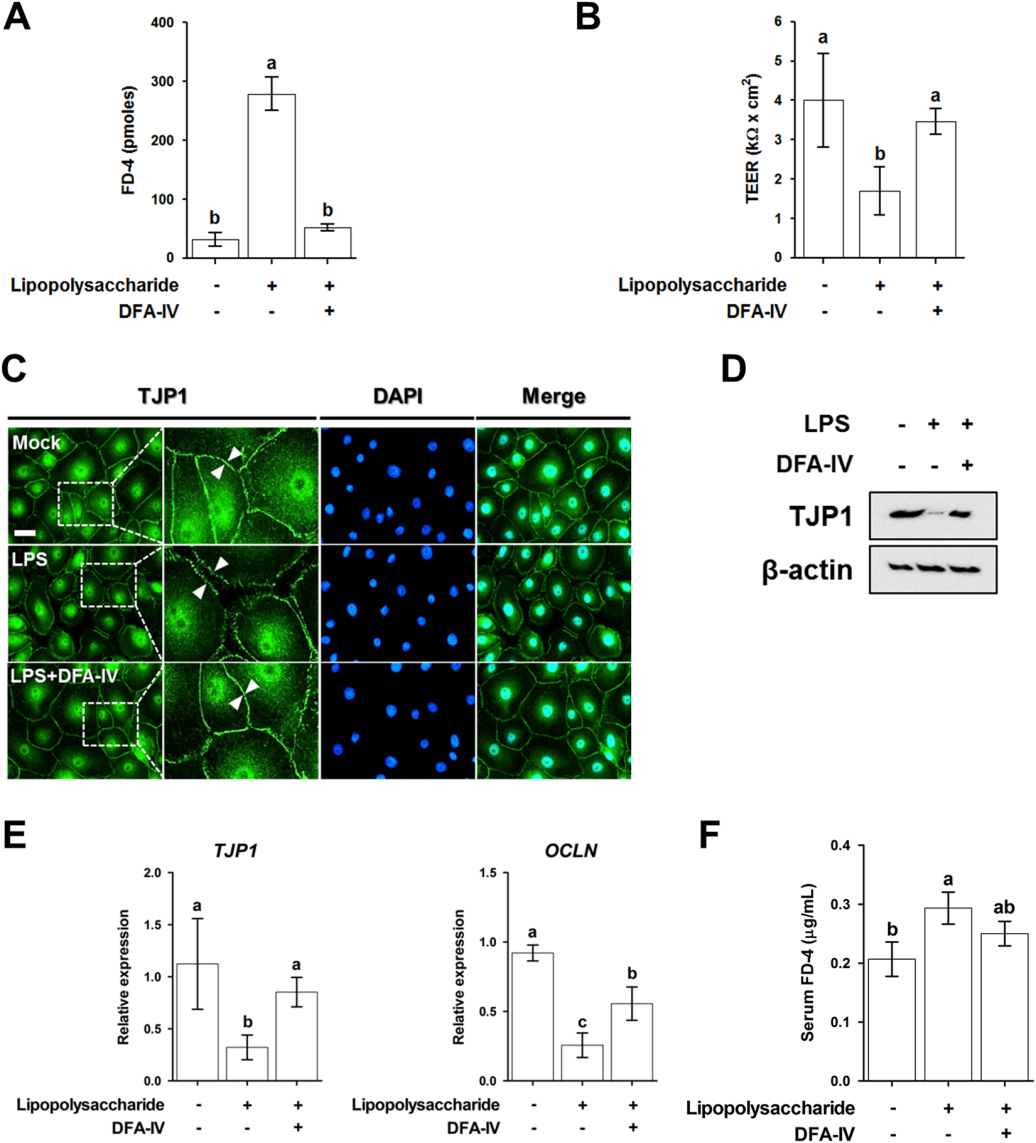
Protective effects of difructose anhydride (DFA)-IV on intestinal barrier function following lipopolysaccharide (LPS) challenge. (**A**) Treatment with DFA-IV (50 μM) increased transepithelial-electrical resistance (TEER) following LPS challenge in IPEC-J2 cells (n = 3). (**B**) Treatment with DFA-IV (50 μM) decreased permeability of fluorescein isothiocyanate-labelled dextrans of 4 kDa (FD-4) following LPS challenge in IPEC-J2 cells (n = 3). Immunofluorescence staining (**C**) and western blotting (**D**) showing the effects of DFA-IV on the expression of tight junction protein 1 (TJP1) in LPS-challenged IPEC-J2 cells. Nuclei were stained with DAPI and arrowhead indicates TJP1 in IPEC-J2 cells. (**E**) Relative quantitative expression of genes encoding tight junctions (*TJP1* and *OCLN*) for DFA-IV (50 μM) treatment after LPS treatment in IPEC-J2 cells. Quantitative reverse transcription polymerase chain reaction (qRT-PCR) data were normalised to expression of *GAPDH* as an endogenous control gene and calculated using the 2^−ΔΔCt^ method. Error bars indicate the standard error of the mean (n = 3); p < 0.05, statistical significance. (**F**) Supplementation of DFA-IV (0.1%) decreased permeability of fluorescein isothiocyanate-labelled dextrans of 4 kDa (FD-4) following LPS challenge in serum of broiler (n = 5).^a,b^Significant differences between treatments using Duncan’s multiple range test. *GAPDH*, glyceraldehyde 3-phosphate dehydrogenase; *TJP1*, tight junction protein-1; *OCLN*, occludin.

To determine whether DFA-IV treatment affects the intestinal barrier function *in vitro*, the TEER and permeability of FD-4 were examined in IPEC-J2 cells. The permeability of FD-4 in LPS-challenged IPEC-J2 cells was significantly higher than that of the control cells (p < 0.05, Fig. 10A). Pre-treatment with DFA-IV substantially reduced the permeability of FD-4 in LPS-challenged IPEC-J2 cells. The TEER in LPS-challenged IPEC-J2 cells was significantly lower than that of the control cells was (p < 0.05, Fig. 10B). Pre-treatment with DFA-IV markedly enhanced the TEER in LPS-challenged IPEC-J2 cells. Next, we investigated whether DFA-IV affects tight junction complexes in LPS-challenged IPEC-J2 cells. The results of immunocytochemistry (Fig. 10C) and western blotting assay (Fig. 10D) demonstrated that TJP1expression level was significantly decreased in IPEC-J2 cells treated with LPS compared with that in control cells and that treatment with DFA-IV in LPS-challenged cells preserved the expression of TJP1. The mRNA expression levels of tight junction protein-1 (*TJP-1*) and *OCLN* were significantly lower in LPS-challenged IPEC-J2 cells than they were in the control cells (p < 0.05, Fig. 10E). Pre-treatment with DFA-IV significantly increased the expression of *TJP-1*and *OCLN* in LPS-challenged IPEC-J2 cells (p < 0.05).

To confirm whether DFA-IV treatment affects the intestinal barrier function *in vivo*, the permeability of FD-4 was examined by determining its levels in the serum of broilers. The permeability of FD-4 based on its serum levels in LPS-challenged broiler was significantly higher than that of the control (p < 0.05, Fig. 10F). Supplementation with DFA-IV did not affect the permeability of FD-4 in LPS-challenged broiler.

## Discussion

The present study found that DFA-IV treatment significantly enhanced ADG, FCR, and relative breast muscle weight in broilers. There is currently no available information on the effect of dietary supplementation with DFA-IV on the growth performance of broilers. However, a previous study demonstrated that dietary supplementation with DFA-enriched caramel increased the body weight of broilers^15^. In this study, we used DFA-IV produced by levan fructan, which contains β-2,6 fructosyl-fructose linkages and exhibits a variety of nutritional and pharmacological effects including the promotion of the ADFI, absorption of metallic ions, antitumor, and immunomodulatory^16–18^. In addition, DFAs stimulated the growth of *Ruminococcus sp*. M-1 by producing acetic acid, which may alter the intestinal microbiota^12^. DFA-IV produced from levan fructan likely stimulated the intestinal microbiota to improve intestinal health, which would ultimately affect growth performance and relative breast muscle weight of broilers. This observation confirmed the expression pattern of genes such as those related to growth factor receptors and muscle development. In addition, we found that digestibility and blood concentration of calcium and iron were increased by dietary supplementation with DFA-IV, confirming the expression pattern of genes such as calcium transport, ATPase, and calcium channel. In agreement with the present study, many previous studies demonstrated that DFAs promote calcium and iron absorption in the intestine, affecting the cecal mucosa to maintain divalent metal transporter-1 (DMT-1) mRNA expression. DMT-1 is a well-known major iron transporter in the small intestinal brush-border membrane and small intestine and is known to play a major role in iron absorption^5,7,19^.

Growth performance is influenced by several biological mediators such as growth hormones and factors. It has been reported that *GH*, *IGF-1*, *IGF-2*, and *LEP* are important gene regulators of growth and body compositions^20–22^. This study revealed that DFA-IV treatment increased the expression levels of *IGF1R*, *IGF2R*, *GHR*, and *LEPR* in the liver. It is well documented that IGFs produced in the liver are important positive modulators of the body and muscle growth. *IGF-1* is one of the main growth factors that stimulate amino acids uptake and incorporation into protein; uridine and thymidine synthesis into nucleic acid; glucose uptake; cell proliferation; and the suppression of protein degradations in muscle tissue^23^. It has been reported that leptin is predominantly expressed in adipose tissues as well as in the liver, the main site of lipogenesis. The liver plays an important role in programming offspring performance^20,24^. *GH* plays a role in postnatal growth enhancement. It regulates metabolism in the liver, adipose tissue, immune system, reproductive system, and cardiovascular system^22,25^. However, a direct relationship between DFA-IV supplementation and increased levels of *IGF1R*, *IGF2R*, *GHR*, and *LEPR* expression in the liver is unclear. The DFA-IV supplementation increased the expression levels of *IGF1R*, *IGF2R*, *GHR*, and *LEPR* in this study probably because DFA-IV supplementation improved calcium and iron digestibility and blood concentration. However, further work is required to determine the direct relationship between DFA-IV supplementation and the expression levels of *IGF1R*, *IGF2R*, *GHR*, and *LEPR* in the liver of broilers.

Intestinal wound healing is a repair process of mucosal damage caused by toxic luminal substances, normal digestion, inflammation, interactions with microbes, oxidative stress, and pharmaceuticals^26^. It is dependent on the precise balance of migration, proliferation, and differentiation of epithelial cells neighbouring the wounded area^26,27^. First, epithelial cells adjacent to the wounded area rapidly migrate to the denuded area to restore barrier integrity in the mucosal damage. Second, migrated epithelial cells proliferate to increase the pool of enterocytes. Third, epithelial cells mature or differentiate to maintain the mucosal barrier function. To investigate the effect of DFA-IV *in vitro*, we used the characterised IPEC-J2 pig intestinal epithelial cell line, which eliminated the need to characterise the primary chicken intestinal epithelial cells, since no commercial chicken intestinal epithelial cell lines are available. This study demonstrated that exogenous DFA-IV treatment induced intestinal epithelial cell proliferation and migration, and increased *MMP2*, *MMP9*, *CDH1*, and *RND3* expression following LPS challenge, inducing wound healing. To date, there is no information on the effect of DFAs on the wound healing (cell migration, proliferation, and differentiation) in the GIT. However, we could infer that DFA-IV affects wound healing based on a previous report that DFA-III increased the depth and number of cells in GIT crypts^28^. To the best of our knowledge, this is the first study to report that treatment with DFA-IV improved intestinal wound healing *in vitro* and *in vivo*.

Intestinal epithelial cells have many functions such as the absorption of nutrients from the lumen by a transcellular permeability mechanism and restriction of the passage of potentially harmful bacteria or toxins by paracellular permeability mechanism as an intestinal epithelial barrier function^29,30^. To maintain effective intestinal epithelial barrier function, it is important that effective intercellular mechanisms such as tight junctions, adherens junctions, and desmosomes that regulate the paracellular permeability of harmful microorganisms and toxins are maintained in intestinal epithelial cells^31^. Among epithelial intercellular junctions, tight junctions play a role in sealing the paracellular space, whereas adherens junctions and desmosomes are critical for the maintenance of the proximity between epithelial cells via intercellular molecular connections^32^. This study found that LPS challenge increased the permeability of fluorescent dextran (40 kDa), whereas DFA-IV treatment restored the impaired epithelial barrier function in the small intestinal epithelial cells. These findings suggest that crosstalk occurs between nutrients and the epithelial barrier function by the dynamic regulation of the tight junction and enhance intestinal barrier function mediated by DFA-IV.

In the present study, DFA-IV modulated the main intestinal functions such as wound healing and permeability under LPS-induced intestinal dysfunction *in vitro*. In addition, the present results indicated that DFA-IV supplementation improved serum calcium and iron concentrations confirmed by calcium transport, ATPase, and calcium channel-related related gene expression. On the basis of these results, we inferred that DFA-IV plays a critical role in the maintenance of the main intestinal functions, which would ultimately affect growth performance and relative breast muscle weight of broilers *in vivo*. Additionally, DFA-IV improved growth performance and relative breast muscle weight, as confirmed by the gene expression of growth-related genes, *IGF1R*, *IGF2R*, *GHR*, and *LEPR* in the liver and muscle-related genes *MSTN*, *MYOD1*, *MYF5*, and *MYOG* in the breast muscle. However, further studies are needed to determine whether DFA-IV directly increased the expression of growth-related genes and thereby enhanced growth performance or improved the growth performance, leading to the increased expression of growth-related genes.

In conclusion, we found that dietary supplementation with DFA-IVs improved growth performance, relative breast muscle and liver weight, digestibility, and blood concentration of calcium and iron in broilers. We also discovered that dietary supplementation with DFA-IVs increased the expression of genes related to growth in the liver, development of the muscle, and absorption of calcium and iron in the intestines. The *in vitro* experiments revealed that DFA-IV treatment improved intestinal wound healing (migration, proliferation, and differentiation) after LPS challenge in the small intestinal epithelial cells. Further, DFA-IV treatment enhanced the intestinal barrier function, thereby increasing the transepithelial electrical resistance (TEER) and decreasing the permeability of FD-4 after LPS challenge in small intestinal epithelial cells. Collectively, these data indicate that DFA-IV could be used as a feed additive to enhance calcium and iron absorption by modulating intestinal wound healing and permeability. This study may improve our understanding of the molecular mechanisms of the effects of DFA-IV on the intestine.

## Methods

### Animals and feeding trial

The animal care and experimental protocols of the present study were approved by the Animal Care, and Use Committee of Dankook University and all methods were performed in accordance with the relevant guidelines and regulations. The DFA-IV produced by levan fructotransferase was kindly provided by the Realbioteck Co., Ltd. A total of 816 broiler chicks (1-d-old male Ross 308) with average body weight (BW) of 43.7 ± 0.34 g were randomly allotted to four experimental diet groups according to their initial BW for a 35-d trial. The following four corn-soybean meal based dietary treatments were used: CON, corn-soybean meal-based control; 0.01% DFA-IV, corn-soybean meal-based control plus 0.01% DFA-IV; 0.05% DFA-IV, corn-soybean meal-based control plus 0.05% DFA-IV; and 0.1% DFA-IV, corn-soybean meal-based control plus 0.1% DFA-IV. All diets were formulated to meet or exceed the nutritional requirements of broilers recommended by *the Nutrient Requirements of Poultry* (Supplemental Table 1)^33^. There were 12 replicate pens per treatment with 17 broilers per pen. All birds were housed in stainless steel pens (1.75 m × 1.55 m). The room temperature was maintained at 33 ± 1 °C for the first 3 d and decreased to 24 °C until the end of the experiment. Diets were fed in two phases (phase I and II from d 0 to 21 and d 21 to 35, respectively). Chicks were allowed free access to water and mash feed during the entire experiment period.

### Growth performance, relative organ weight, nutrient digestibility, and blood concentration of calcium and iron

BW, feed intake, and feed conversion per cage were recorded on d 0, 21, and 35 (the end of the experiment). The FCR was calculated as the feed intake divided by body weight gain. The broilers in the various treatments groups (n = 10) were weighed individually and euthanised by cervical dislocation. Then, the liver and breast meat were removed and weighed. The relative organ weights were expressed as a percentage of the live body weight.

At the end of the experiment, the nutrient digestibility of calcium and iron were determined using chromic oxide (Cr_2_O_3)_ as an indicator. Briefly, all broiler chicks were fed diets mixed with 2% Cr_2_O_3_ for 7 d before excreta collection at week 4. All excreta were pooled by pen and mixed. A representative sample was stored in a freezer at −20 °C until the analysis. Before chemical analysis, the excreta samples were thawed and dried at 50 °C for 72 h in a forced-air oven (model FC-610, Advantec, Toyo Seisakusho Co. Ltd., Tokyo, Japan). After drying, the samples were finely ground to a size that could pass through a 1 mm screen. The calcium and iron of all feed and faecal samples were then analysed using ultraviolet (UV) absorption spectrophotometry (UV-1201, Shimadzu, Kyoto, Japan).

At the end of the experiment, blood samples from each treatment group (n = 10) were collected from the jugular vein into tubes containing dipotassium ethylenediaminetetraacetic acid (K_2_EDTA, BD Vacutainer, Plymouth, PL6 7BP, UK), centrifuged at 3000 × *g* for 15 min, and then stored at −80 °C. Blood calcium and iron concentration were estimated using an automatic blood analyser (ADVIA 120, Bayer, NY, USA).

### RNA extraction and quantitative reverse transcription polymerase chain reaction (qRT-PCR)

Total RNA was isolated from the breast muscle, liver, and small intestine (duodenum, jejunum, and ileum) using TRIzol reagent (Invitrogen, CA, USA) at the end of the experiment. For the quantitative reverse transcription polymerase chain reaction (qRT-PCR), total RNA (1 µg) was used for cDNA synthesis using the Maxima First Strand cDNA synthesis kit (Life Technologies, CA, SA). Primers for each gene transcript used for the qRT-PCR were designed using Primer3 program (http://frodo.wi.mit.edu/). The primers used in this study are listed in Supplemental Table 2. The qRT-PCR analysis was performed using a 7500 Fast Real-Time PCR system (Applied Biosystems, CA, USA). The qRT-PCR conditions were: 94 °C for 3 min followed by 40 cycles at 94 °C for 30 s, 59–61 °C for 30 s, and 72 °C for 30 s. Melting curve profiles were analysed for the amplicons. The qRT-PCR data were normalised to the expression of glyceraldehyde 3-phosphate dehydrogenase (*GAPDH*) as the endogenous control. The gene expression was calculated using the 2^ΔΔCt^ method, where ΔΔ^Ct^ = (Ct of the target gene − Ct of *GAPDH*) of the treatment − (Ct of the target gene − Ct of *GAPDH*) of the control^34^.

### Porcine intestinal epithelial cell culture

The non-transformed porcine intestinal epithelial cell line (IPEC-J2, DSMZ, Braunschweig, Germany), originally isolated from the jejunal epithelia of a neonatal unsuckled piglet, were cultured in Dulbecco’s modified Eagle’s medium (DMEM) and Ham’s F-12 medium mixed at a 1:1 ratio (Gibco Life Technologies, Grand Island, NY, USA) supplemented with 5% foetal bovine serum, 1% insulin-transferrin-selenium-X, and 1% (v/v) penicillin-streptomycin mixture. Cells were grown at 37 °C in a humidified atmosphere of 5% CO_2_. IPEC-J2 cells were incubated with various concentrations of DFA-IV for 24 h before LPS stimulation. For cyclophosphamide induction, the IPEC-J2 cells were incubated with 1 μg/μL LPS for 1 h, and the LPS was removed by washing twice with phosphate-buffered saline (PBS).

### Cell proliferation and migration assay

IPEC-J2 cells were seeded in 96-well plates at a density of 1 × 10^4^ cells/well. The cell proliferation reagent, water-soluble tetrazolium (WST)-1 (Roche Applied Science, Indianapolis, IN, USA) was added to each well according to the manufacturer’s instructions. The absorbance of the dye-treated reaction solution after incubation was measured at a wavelength of 450 nm with background subtraction at 690 nm using a BioTek Synergy HTTR microplate reader (BioTek Instruments, Winooski, VT, USA). IPEC-J2 cells were cultured with or without DFA-IV in 60 mm culture dishes until they reached confluence. Subsequently, straight scratches were created using a p200 pipette tip across the 60-mm culture dish, simulating a wound. Images were acquired at different intervals as indicated in the figure legends.

### Transepithelial electrical resistance and intestinal permeability

IPEC-J2 cells were grown to confluent monolayers (≥1 kΩcm²) in 24-well Corning Transwell chambers (polycarbonate membrane, filter pore size 0.4 μm, area 0.33 cm^2^; Costar) and then treated with or without DFA-IV for 24 h. The cells were washed twice and incubated with LPS for 1 h. The TEER was measured using an epithelial volt-ohm meter (World Precision Instruments, Sarasota, FL, USA). TEER values were calculated by subtracting the blank filter (90 Ω) and multiplying the surface area of the filter. All measurements were performed on a minimum of triplicate wells.

The permeability assay was initiated by adding 500 μL culture medium containing 1 mg/mL FD-4 (Sigma-Aldrich) was added to the apical chamber. The basolateral chamber was filled with 1.5 mL culture medium (37 °C, 5% CO_2_). FD-4 was allowed to permeate overnight (18 h) from the apical to the basolateral chamber. Subsequently, 100 μL of the basolateral chamber medium was transferred to a 96-well plate to measure the amount of permeated FD-4 using a fluorospectrophotometer (Ex/Em: 490/520 nm).

For *in vivo* intestinal permeability assay, total 15 chickens (10 chicken for CON and 5 chickens for 0.1% DFA-IV) were weighed and injected intraperitoneally with LPS or saline solution (0.1 g/kg) on d 10 at 4 h after LPS challenge; 5 chickens per treatment were orally gavaged with FD-4 (4.16 ug/mL). Blood serum samples were obtained 2.5 h post-gavage, and 100 μL serum samples were transferred to a 96-well plate to measure the amount of permeated FD-4 using a fluorospectrophotometer (Ex/Em: 490/520 nm).

### Statistical analysis

The data were statistically analysed using an analysis of variance (ANOVA) with a general linear model (GLM) procedure of the SAS program (SAS Institute, NC), using a completely randomised design. The data were presented as means ± standard error of the means. To determine the significance of treatments, the data were analysed using the GLM using the SAS program. Differences among all treatments were analysed using Duncan’s multiple range tests. A p < 0.05 was considered statistically significant. To determine the significance between treatment and control groups, data were analysed using the Student’s *t*-test with the SAS software for qRT-PCR analysis. Significant differences between the control and treatment groups are indicated as *p < 0.05, **p < 0.01, and ***p < 0.001.

## Electronic supplementary material

Supplemental information
