# Supplementary material for: Difructose dianhydride improves intestinal calcium absorption, wound healing, and barrier function
Source: Sci Rep. 2018 May 18;8:7813. doi: 10.1038/s41598-018-26295-7 (PMC5959885; doi:10.1038/s41598-018-26295-7)
Supplement: Supplementary file 1 — Supplemental information [file 41598_2018_26295_MOESM1_ESM.pdf]

Difructose dianhydride improves intestinal calcium absorption, wound healing, and barrier function

Sang In Lee and In Ho Kim *

Whole blot image of Figure 10D.

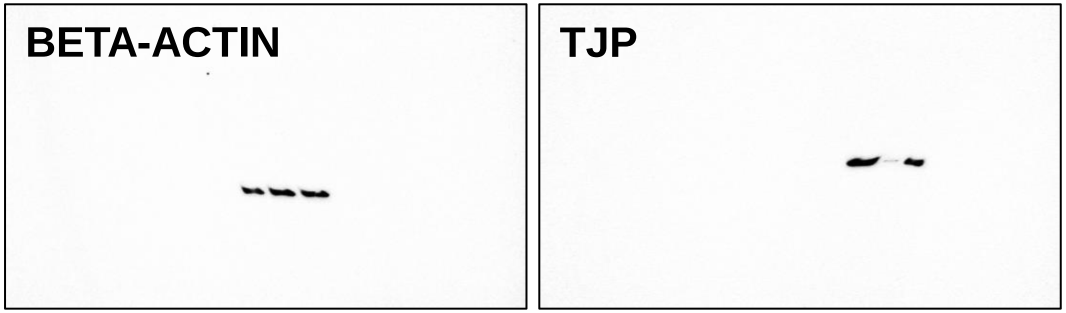

Difructose dianhydride improves intestinal calcium absorption, wound healing, and barrier function

Sang In Lee and In Ho Kim *

Supplemental table 1. Composition of control diet (as-fed-basis)

Item	Phase I	Phase II
**Ingredients (%)**		
Corn	56.95	60.44
Soybean meal, 45% CP	29.25	25.33
Corn gluten meal, 60% CP	4.44	3.83
Tallow	3.61	5.00
Limestone	0.91	1.02
Dicalcium phosphate	2.07	1.93
Salt	0.32	0.37
DL-Methionine	0.33	0.37
L-Lysine-HCI	1.68	1.28
L-Threonine	0.18	0.18
Choline chloride	0.10	0.10
Vitamin premix[1]	0.06	0.05
Trace mineral premix[2]	0.10	0.10
**Calculated Composition**		
Metabolizable energy, kcal/kg	3,050	3,200
Crude protein, %	21.00	19.00
Lysine, % %	1.40	1.20
Calcium, %	0.90	0.90
Total Phosphorus, %	0.71	0.66

1Provided per kg of complete diet: vitamin A, 11,025 IU; vitamin D3, 1,103 IU; vitamin E, 44 IU; vitamin K, 4.4 mg; riboflavin, 8.3 mg; niacin, 50 mg; thiamine, 4 mg; pantothenic acid, 29 mg; choline, 166 mg; and vitamin B12, 33 μg
2Provided per kg of complete diet: Cu, 12 mg; Zn, 85 mg; Mn, 8 mg; I, 0.28 mg; and Se, 0.15 mg

Supplemental table 2. list of primers

Gene symbol	Description	Acession No.	Primers (5' --> 3')	
			Forward	Reverse
SLC8A1	solute carrier family 8 (sodium/calcium exchanger), member 1	NM_001079473	TCGTACCCCCGACAGACTAC	GCCACCTTACTGGCAAATGT
SLC8B1	solute carrier family 8 (sodium/lithium/calcium exchanger), member B1	XM_015294864	CTTCGAGCTGAGCAACACTG	CCCACACCCACCAGAATATC
SLC24A1	solute carrier family 24 (sodium/potassium/calcium exchanger), member 1	NM_001001773	AACAGCCTTACAGCGAGGAA	CTGGACGGTGTTTTGTGATG
ATP2A1	ATPase, Ca++ transporting, cardiac muscle, fast twitch 1	NM_205519	CGCTGTCAATCAGGACAAGA	GTCGTTAAAGTGGCCGATGT
ATP2B1	ATPase, Ca++ transporting, plasma membrane 1	NM_001168002	CTGGGCATGGGAACACTACT	CACGACGTAATTCTCGCTCA
ATP13A4	ATPase type 13A4	NM_001031314	CCAAAGCTCCTGCTAAATGC	ATGCCTCCTGCTCTGACAGT
CACNA1A	calcium channel, voltage-dependent, P/Q type, alpha 1A subunit	XM_015273632	GCAGCGGGTCTATAAGCAGT	GCGATGATGGTGGCTAAAAT
CACNB1	calcium channel, voltage-dependent, beta 1 subunit	XM_015299530	GGCAGCATATGGTGTGTGAC	GGACAAAATCCTGGTGCTGT
CACNG1	calcium channel, voltage-dependent, gamma subunit 1	NM_001278006	CAGCTACTCCTGGTCCTTCG	GGAGATCCCGACATCTGAAA
TPCN1	two pore segment channel 1	XM_004934492	GACGGCCTGTCTCTGAGTTC	ATCCCACTGCCTGATTTACG
TPCN2	two pore segment channel 2	XM_004941528	AGGTGCTGTGGTTCCTATGG	GCCCACGGACTTGTGTATCT
TPCN3	two-pore calcium channel 3	NM_001143931	TGGGAATGGGAGTTCAAGAG	CTGCCTCAAACATACGCTGA
CASR	calcium sensing receptor	XM_416491	CTGCGTGATTTGGCTCTACA	GGCAAAGAAGAAGCAGATGG
IGF1R	insulin like growth factor 1 receptor	NM_205032	AGCATTAAGGACGAGCTGGA	TCCTGAGTGCTTGTCAGTGG
IGF2R	insulin like growth factor 2 receptor	NM_204970	AAAGAATAGGCCGTGGTGTG	GCAGCCAAGCATATCACTCA
LEPR	leptin receptor	NM_204323	CCCCAAGACTCAACCACACT	ACAGGCTCAGACCAGCAGAT
GHR	growth hormone receptor	NM_001001293	CTTCAGTGCAAGCGACACAT	GGCCATGACTCTCTGCTTTC
MSTN	myostatin	NM_001001461	CAGGACCGGGTGAAGATGGA	GGGTAGCGACAACATCGGGA
MYOD1	myogenic differentiation 1	NM_204214	GGCCGCCGATGACTTCTATG	TGCTCCTCCTCGTGTGGGTT
MYOG	myogenin (myogenic factor 4)	NM_204184	AGCGATGACCAGGCAGAGGA	CCAGCTCAGTTTTGGACCCG

MYF5	myogenic factor 5	NM_001030363	AGGATTTCGAGCCCAGGGAG	TCATAGTGGCTGCCTTCCGC
MMP2	matrix metallopeptidase 2	NM_204420	GCTATACCCCGGATTTGGAT	CAAAATGGGAGTCTCCTCCA
MMP9	matrix metallopeptidase 9	NM_204667	GCTACATCCAGGAGGCAGAG	TCGGGGGAGTAGTTCATCAC
CDH1	cadherin 1	NM_001039258	TGTACGAAGGTGTGGTGGAA	AGCGGCTCTTGGTCTCATAA
CDX2	caudal type homeobox 2	NM_204311	TCAAAACCAGGACGAAGGAC	CCAGATTTTCACCTGCCTCT
PCNA	proliferating cell nuclear antigen	NM_204170	GAGACCTCAGCCACATTGGT	AGTCAGCTGGACTGGCTCAT
TP53	tumor protein p53	NM_000546	GGCCCACTTCACCGTACTAA	GTGGTTTCAAGGCCAGATGT
RND3	Rho family GTPase 3	XM_422158	CAACATGTGGGACACCTCAG	ATTTGCAACCAACCAAAAGC
TJP1	tight junction protein 1	XM_015278981	AGGTGAAGTGTTTCGGGTTG	CCTCCTGCTGTCTTTGGAAG
TJP2	tight junction protein 2	NM_204918	GAAAGCTCCAGCTGGTTGTC	TGGATGAATGCAAATCCAGA
TJP3	tight junction protein 3	XM_015299757	CCATGGTGGTGTTCTGTGAG	TGAGGCTGATGGTAGCAGTG
OCLN	occludin	NM_205128	GTCTGTGGGTTCCTCATCGT	GTTCTTCACCCACTCCTCCA
GAPDH	glyceraldehyde-3-phosphate dehydrogenase	NM_204305	GGTGGTGCTAAGCGTGTTAT	ACCTCTGTCATCTCTCCACA
